# The effects of the heme precursor 5-aminolevulinic acid (ALA) on REV-ERBα activation

**DOI:** 10.1016/j.fob.2014.03.010

**Published:** 2014-03-26

**Authors:** Kohei Yamashita, Yuichiro Hagiya, Motowo Nakajima, Masahiro Ishizuka, Tohru Tanaka, Shun-ichiro Ogura

**Affiliations:** a Graduate School of Bioscience and Biotechnology, Tokyo Institute of Technology, 4259-B102, Nagatsuta-cho, Midori-ku, Yokohama 226-8501, Japan; b SBI Pharmaceuticals CO., LTD., Izumi Garden Tower 20F, 1-6-1, Roppongi, Minato-ku, Tokyo 106-6020, Japan

**Keywords:** ALA, 5-aminolevulinic acid, Ct, cycle threshold, DMEM, Dulbecco’s modified Eagle’s Medium, HPLC, high-performance liquid chromatography, PpIX, protoporphyrin IX, SFC, sodium ferrous citrate, 5-Aminolevulinic acid, Circadian rhythms, REV-ERBα, Porphyrin metabolism

## Abstract

The nuclear receptor, REV-ERBα, has a key role in circadian rhythms and requires heme as its ligand. The present study determined whether the heme precursor, 5-aminolevulinic acid (ALA), affects REV-ERBα and its target genes. When exposed to ALA, the human lung diploid cell line, WI-38, exhibited activation of REV-ERBα and repression of the transcription of REV-ERBα target genes, including *BMAL1*, an essential component of the circadian oscillator. Moreover, co-incubation of sodium ferrous citrate (SFC) and ALA also activated REV-ERBα and repressed the transcription of REV-ERBα target genes. These results indicate that ALA regulates human circadian rhythms via REV-ERBα.

## Highlights

• REV-ERBα has a key role in circadian rhythms and requires heme as its ligand.
• Heme precursor ALA exhibited increased nuclear heme level and activation of REV-ERBα.
• ALA inhibited REV-ERBα target genes including an essential component of the circadian oscillator.

## 1 Introduction

Humans have circadian rhythms that allow them to adapt to environmental change caused by the earth’s rotational period [1–3]. The suprachiasmatic nucleus in the hypothalamus is known to control the central clock in mammalian cells. Many tissues have circadian rhythms referred to as peripheral clocks in addition to a central clock [4]. Circadian rhythm is controlled by feedback loops consisting of transcription and translation of clock proteins and is mainly regulated by loops consisting of the heterodimeric protein complex of BMAL1/CLOCK, BMAL1/NPAS2, and PER/CRY. The complex of BMAL1/CLOCK and BMAL1/NPAS2 activate transcription of clock genes, including *PER* and *CRY*, by binding E-box [5,6]. These transcriptional products form the complex and suppress the activities of BMAL1/CLOCK and BMAL1/NPAS2 [2,4]. REV-ERBα and REV-ERBβ, typical nuclear receptors, strongly modulate circadian rhythms by binding to the ROR region of *BMAL1* or *CLOCK* and suppresses the expression of these genes [7]. Heme was reported to be a ligand of REV-ERBα and REV-ERBβ and found to be fundamentally important for their activities [8,9].

5-Aminolevulinic acid (ALA) is widely distributed in both plants and animals and is the common precursor of heme [10]. ALA administration induces cancer-specific accumulation of fluorescent protoporphyrin IX (PpIX) [11]. Therefore, fluorescence diagnosis, also termed photodynamic diagnosis, is based on the different ALA-induced fluorescent signatures of normal and cancer tissues [12]. In contrast, in a normal tissue, the iron ion is inserted into PpIX to form heme in the mitochondria and incorporated into hemoproteins. Our previous study showed that the administration of ALA increased cytochrome *c* oxidase activity in mice [13]. These findings strongly suggest that the administration of ALA leads to the accumulation of intracellular heme. The present study tested the possible impact of ALA on the endogenous activities of REV-ERBα *in vitro* to determine whether this compound influences circadian rhythms in human cells. This study demonstrates the potential of ALA as an endogenous compound that affects circadian rhythms.

## 2 Materials and methods

### 2.1 Biochemicals

ALA hydrochloride and sodium ferrous citrate (SFC) were purchased from Cosmo Oil Co., Ltd. (Tokyo, Japan). Dulbecco’s modified Eagle’s Medium (DMEM) and protease inhibitor cocktail and antibiotic–antimycotic solution were obtained from Nacalai Tesque (Kyoto, Japan). Fetal bovine serum (FBS) and horse serum were purchased from Invitrogen (Carlsbad, CA, USA). All other chemicals used were of analytical grade.

### 2.2 Cell culture

Human lung diploid cells (WI-38) were purchased from RIKEN Cell Bank (Tsukuba, Japan). The cells were maintained under 5% CO_2_ gas at 37 °C in DMEM supplemented with 10% (v/v) fetal bovine serum and 1% (v/v) antibiotic–antimycotic solution.

### 2.3 HPLC analysis of PpIX and heme

Cells (1 × 10^6^ cells) were incubated with 1 mM ALA and 0.5 mM SFC in DMEM under 5% CO_2_ gas at 37 °C in the dark each time. Cells were rinsed with 1 ml of phosphate-buffered saline (PBS) and then treated with 300 μ1 of 0.1 M NaOH. An aliquot (10 μl) of the NaOH-treated cell sample was withdrawn and used for protein concentration assay (Quick Start™ Bradford protein assay, Bio-Rad Laboratories, Inc., CA, USA), whereas the remaining cellular proteins were denatured by adding 3-fold volumes of solvent A [l M ammonium acetate, 12.5% acetonitrile (v/v)]: solvent B [50 mM ammonium acetate, 80% acetonitrile (v/v)] (1:9 v/v) solution to the NaOH-treated cell sample. The prepared sample was centrifuged at 12,000 rpm for 10 min at 4 °C and subjected to high-performance liquid chromatography (HPLC) analysis performed as previously described with some modifications [14,15]. In brief, protoporphyrin IX (PpIX) and heme were separated using the HPLC system (Prominence, Shimadzu, Kyoto, Japan) equipped with a reversed-phase C_18_ column (CAPCELL PAK, C18, SG300, 5 μm, 4.6 mm × 250 mm, Shiseido, Tokyo, Japan). Elution was started with 10% solvent A and 90% solvent B for 7 min. The elution flow throughout was kept constant at a rate of 2.0 ml/min. PpIX and heme were continuously detected using a spectrophotometer at 404 nm. The concentration of the samples was estimated using calibration curves of reference standards.

### 2.4 Quantitative PCR

A High Pure RNA Isolation Kit (Roche Ltd., Mannheim, Germany) was used to extract total RNAs from the cells according to the manufacturer’s protocol [16,17]. One microgram of total RNAs was reverse-transcripted to produce first strand cDNA using the PrimeScript RT reagent Kit with gDNA Eraser (TaKaRa, Shiga, Japan) according to the manufacturer’s protocol [16,17]. The Thermal Cycler Dice Real Time System (TaKaRa, Shiga, Japan) was used for a 2-step reverse transcription polymerase chain reaction (PCR). The mRNA transcripts were quantified by SYBR Premix ExTaq (TaKaRa, Shiga, Japan). *HO-1* specific primers 5′-GCTCAAAAAGATTGCCCAGAA-3′ and 5′-TCACATGGCATAAAGCCCTACA-3′ were used. The other primers were purchased from TaKaRa. The amplification conditions included 30 s at 95 °C, a run of 45 cycles at 95 °C for 5 s, and 60 °C for 60 s followed by dissociation for 15 s at 95 °C and 30 s at 60 °C and then 15 s at 95 °C on the Thermal Cycler Dice Real Time System. The Thermal Cycler Dice Real Time System analysis software (TaKaRa, Shiga, Japan) was used to analyze the data. The Ct values (cycle threshold) were calculated by the crossing-point method, and the relative quantities of target mRNA expression levels were measured by the comparison of standard curve. The results for each sample were normalized to *ACTB*, the housekeeping gene.

### 2.5 Western blot analysis

Western blot analysis was performed as previously described with some modifications [18]. Cells were lysed in whole-cell lysis buffer A [50 mM Tris–HCl (pH 7.4), 1 mM DTT, 1% (v/v) Triton X-100, and protease inhibitor cocktail (Nacalai Tesque, Kyoto, Japan)]. Sodium dodecyl sulfate polyacrylamide gel electrophoresis (SDS–PAGE) was used to separate 20 μg of lysates, which were then transferred to polyvinylidene difluoride membranes. The following primary antibodies were used to probe the blots: anti-ALAS1 (Abcam, Cambridge, UK, ab54758), anti-BMAL1 (Abcam, Cambridge, UK, ab3350, [19]), anti-HO-1 (Abcam, Cambridge, UK, ab13248, [20]), anti-NR1D1 (Abcam, Cambridge, UK, ab56754, [21]), anti-ACTIN (MP Biomedicals, Irvine, CA, USA, 69100), and anti-HDAC3 (Cell Signaling Technology, Beverly, MA, USA, 3949). For the second antibody, we used anti-mouse IgG HRP-conjugated antibody (Cell Signaling Technology, Beverly, MA, USA) and anti-rabbit IgG HRP conjugates (Santa Cruz Biotechnology) at 1:3000 dilution.

### 2.6 Nuclear extraction

An EpiQuik Nuclear Extraction Kit II (Epigentek Group Inc., Brooklyn, NY, USA) was used to isolate nuclear extracts according to the manufacturer’s protocol. HPLC and Western blot analysis were used to determine heme accumulation and protein expression levels, respectively.

### 2.7 Serum stimulation

To synchronize circadian rhythms, serum stimulations were performed [22]. Cells (5 × 10^6^ cells/well) were pre-incubated with or without 1 mM ALA and 0.5 mM SFC in DMEM supplemented with 10% FBS for 4 h. Then cells were collected as zero-point and started the collection as below. In the first 2 h, cells were stimulated by changing the medium to serum-rich (50% (v/v) horse serum) DMEM to synchronize circadian rhythms with or without 1 mM ALA and 0.5 mM SFC; this medium was then replaced with serum-free DMEM with or without 1 mM ALA and 0.5 mM SFC after 2 h. In ALA and SFC addition group, 1 mM ALA and 0.5 mM SFC were re-added after 24 h. At the indicated times after serum stimulation, the cells were washed twice with 1 ml of PBS. The cells were collected for RNA extraction at the indicated time points.

## 3 Results

**Fig. 1:**
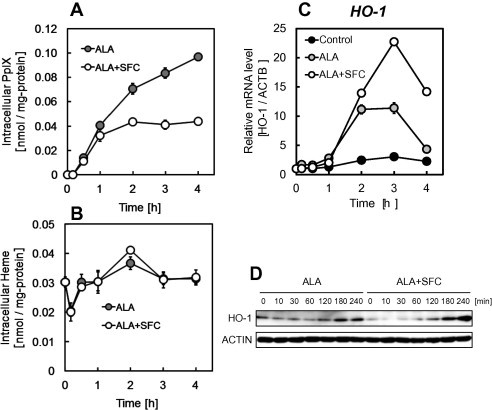
Intracellular PpIX (A) and heme (B) accumulation and relative expression levels of *HO-1* mRNA (C) and HO-1 protein (D) in WI-38 cells. Cells were incubated with 1 mM ALA and 0.5 mM SFC (white) or ALA alone (gray). PpIX and heme accumulation were determined by HPLC analysis. Expressions of *HO-1* mRNA and proteins were determined by real-time PCR analysis and Western blot analysis respectively. Results represent the mean ± SD.

In the present study, to accumulate intracellular heme, ALA was administered. Because heme is formed by the insertion of the iron ions into PpIX converted from ALA, the effect of administration of SFC (used as an iron ion source) was investigated. Fig. 1A shows intracellular PpIX accumulation after incubation with ALA and SFC or ALA alone. WI-38 cells were used in this study. Intracellular PpIX accumulation was observed by ALA incubation; however, PpIX accumulations were decreased by ALA/SFC co-incubation. This decreased PpIX was converted into heme by co-incubation of ALA and SFC. The intracellular heme level was measured using HPLC (Fig. 1B). Heme accumulation was slightly observed 2 h after ALA/SFC co-incubation; however, significant changes were not observed. Heme is known to be degraded by HO-1 immediately; therefore, HO-1 levels were investigated after ALA and SFC incubation. Fig. 1C shows mRNA expression levels of *HO-1*, and Fig. 1D shows protein expression levels of HO-1 measured using Western blotting after ALA and SFC incubation showing HO-1 levels were increased in ALA incubation and ALA/SFC co-incubation. These results indicate that heme was accumulated and degraded by HO-1. Furthermore, HO-1 level was higher in ALA and SFC co-incubation than in ALA incubation only, which indicated that heme accumulation in ALA and SFC co-incubation were higher than that in ALA incubation.

**Fig. 2:**
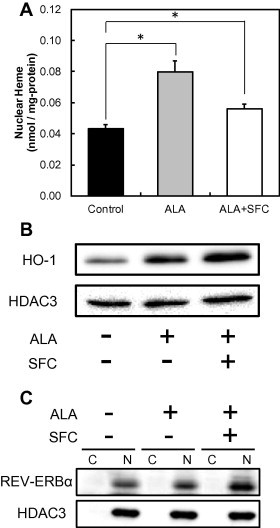
Heme accumulation (A), HO-1 protein level (B) and RER-ERBα protein level (C) in nuclear fraction from WI-38 cells. REV-ERBα protein level in cytoplasmic (C) and nuclear (N) fractions were determined. Cells were incubated with 1 mM ALA and 0.5 mM SFC or ALA alone for 2 h. Results represent the mean ± SD. Statistical significance of difference is indicated by ^∗^*p* < 0.01 as compared between paired groups. Statistical significance was calculated using Student’s *t*-test.

REV-ERBα functions after binding to heme as a ligand in the nucleus. Therefore, HPLC was used to determine intranuclear heme. Fig. 2A shows intranuclear heme concentrations after ALA incubation for 2 h with and without SFC. Intranuclear heme after ALA incubation was significantly increased. Intranuclear heme after ALA/SFC co-incubation was higher than that of the control but lower than that after only ALA incubation. These results indicate that excess heme was degraded by HO-1. In Fig. 2B, HO-1 protein expression levels in nuclear fractions showing apparent inductions of HO-1 are observed. Furthermore, protein expression levels of REV-ERBα in the nucleus were increased by ALA incubation and ALA/SFC co-incubation (Fig. 2C). In Fig. 2C, band intensities were quantified resulting ALA incubation resulted 1.1 times higher than without ALA incubation (control) and ALA/SFC co-incubation resulted 1.6 times higher than control. These results strongly indicate that ALA incubation and ALA/SFC co-incubation leads to heme accumulation in the nucleus and induces REV-ERBα.

**Fig. 3:**
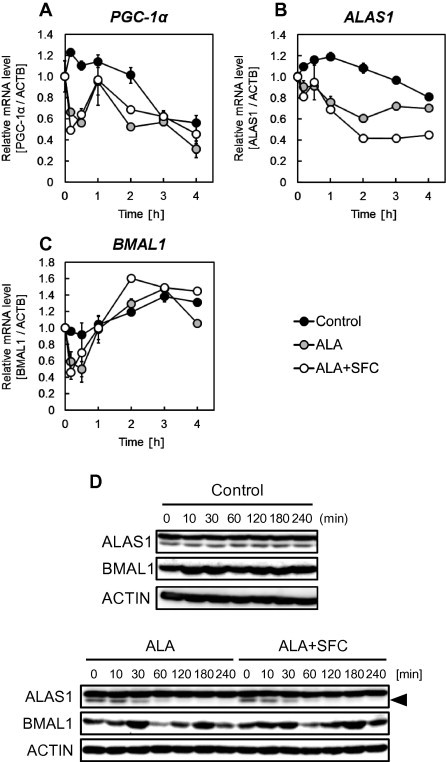
Relative mRNA expression levels of *PGC-1α* (A), *ALAS1* (B), *BMAL1* (C) and protein expression levels (D) in WI-38 cells. Cells were incubated with 1 mM ALA and 0.5 mM SFC (white) or ALA alone (gray). Results represent the mean ± SD.

REV-ERBα is known to regulate the expression of target genes, such as *PGC-1α*, *ALAS1*, and *BMAL1*. The mRNA expression levels of *PGC-1α* (Fig. 3A), *ALAS1* (Fig. 3B), and *BMAL1* (Fig. 3C) were measured using real-time PCR after ALA incubation and ALA/SFC co-incubation. The mRNA expression levels of all 3 genes were suppressed within 1 h. As shown in Fig. 2, the intranuclear heme level increases in 2 h, and heme is degraded by HO-1. This result is in good agreement with the results that target genes of REV-ERBα were suppressed within 1 h. Moreover, the protein expression levels of ALAS1 and BMAL1 were suppressed (Fig. 3D), which showed that REV-ERBα repressed the transcription of target genes.

**Fig. 4:**
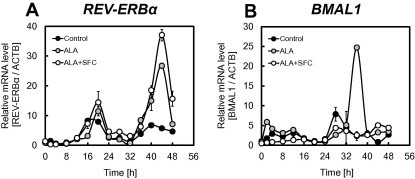
Relative mRNA expression levels of *REV-ERBα* (A), and *BMAL1* (B) after 50% serum shock. Cells were incubated with 1 mM ALA and 0.5 mM SFC (white) or ALA alone (gray). Results represent the mean ± SD.

From the above results, ALA incubation and ALA/SFC co-incubation activated the expression of REV-ERBα and suppressed its target genes, including clock genes. It is well known that serum stimulations cause synchronizing circadian rhythms and that the amplitude and cycle of circadian rhythms could be detected. Fig. 4 shows the effect of ALA incubation and ALA/SFC co-incubation on circadian rhythms after serum stimulations. The expression levels of *REV-ERBα* were increased by ALA incubation and ALA/SFC co-incubation (Fig. 4A). This result is in good agreement with that of Fig. 2C, which shows that REV-ERBα protein level was increased. Furthermore, the expression of *BMAL1*, a typical target gene of REV-ERBα, was suppressed by ALA/SFC co-incubation (Fig. 4B). Because these genes strongly affect circadian rhythms, ALA and ALA/SFC incubation could affect circadian rhythms.

## 4 Discussion

Since the mouse Clock gene was discovered [23], many studies on circadian rhythms have been performed. Recent studies have shown that abnormal circadian rhythms increase the risk for diabetes, bipolar disorder, cancer, and coronary events [24–27]. These results strongly suggest that the shift to normal circadian rhythms from abnormal circadian rhythms is quite important. Kaasik et al. showed that addition of hemin, the Fe(III) form of heme, affected circadian rhythms [28]. However, the addition of hemin leads to increased free-heme and cytotoxicity [29]. From the above results, the administration of heme or hemin to humans is quite difficult.

**Fig. 5:**
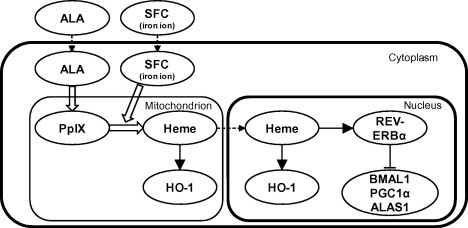
Schematic illustration of the heme synthesis and the activation of REV-ERBα in this study.

ALA is used in various fields, especially cancer diagnosis (photodynamic diagnosis) and cancer therapy (photodynamic therapy) [10]. There are no reports of toxic side effects caused by ALA administration. It is well known that ALA is converted into heme and that heme has an important role in the activation of REV-ERBα. Therefore, in the present study, the effects of ALA on circadian rhythms via REV-ERBα were investigated. ALA incubation led to the activation of heme biosynthesis and suppression of REV-ERBα target genes (Fig. 5). Moreover, these effects were enhanced by ALA/SFC co-incubation, and ALA/SFC co-incubation affected the oscillation and phase of core clock genes. These phenomena were supported by increased heme level in the nucleus. To our knowledge, this is the first attempt to regulate circadian rhythms using ALA.

Because the target gene of REV-ERBα is known to cause several diseases, drug design strategies that modulate REV-ERBα activity are expanding [30–32]. Laura et al. showed that SR9009a and SR9011 acted as strong agonists and modulated several clock genes [32]. Because ALA also modulated REV-ERBα; therefore, ALA could be a new candidate of REV-ERBα modulator that regulates several diseases. However, further research is required to examine the actual contribution of ALA *in vivo* using experimental animal models.
